# Distribution and soil threshold of selenium in the cropland of southwest mountainous areas in China

**DOI:** 10.1038/s41598-024-67450-7

**Published:** 2024-07-23

**Authors:** Sheng Wang, Qi Liu, Zhizong Liu, Wen Chen, Xuanyue Zhao, Jilai Zhang, Li Bao, Naiming Zhang

**Affiliations:** 1 College of Plant Protection, Yunnan Agricultural University, Kunming, 650201 Yunnan China; 2 College of Resources and Environment, Yunnan Agricultural University, Kunming, 650201 Yunnan China; 3 Yunnan Soil Fertility and Pollution Remediation Engineering Research Center, Kunming, 650201 Yunnan China

**Keywords:** Mountainous area, Selenium, Cropland, Influencing factors, Se-rich soil threshold, Agroecology, Environmental sciences

## Abstract

To investigate the distribution characteristics of selenium (Se) in mountainous soil-crop systems and examine the threshold value of Se-rich soil, 275 soil samples and 153 associated crop samples (rice, maize, tea, nuts, konjac, mushrooms, buckwheat, and coffee) were collected in Ximeng County, a typical mountainous area in southwest China. The total Se, available Se, organic matter, pH, sampling point elevation, and crop Se content were analyzed to examine the distribution characteristics of soil Se and the ability of primary crops to enrich Se in Ximeng County. Random forest and multiple regression models were established to identify the factors influencing the available soil Se and the crop Se enrichment coefficient. Finally, the Se-rich soil threshold was examined based on the total Se, available Se, and Se content in primary crops (rice, maize, and tea). The results showed soil Se resource abundance in the study region, with high Se soil accounting for 64.72% of the entire area. The soil Se content displayed significant spatial autocorrelation. The average Se enrichment coefficient of the main cultivated crops included mushrooms > nuts > rice > coffee > tea > maize > buckwheat > konjac. The total Se content in the soil had the highest impact on the available Se content in the soil and the Se enrichment coefficient of crops. A Se-rich soil threshold of 0.3 mg·kg^−1^ was used for rice and maize, while that of tea was 0.4 mg·kg^−1^. This result provided a theoretical basis for developing and utilizing Se resources in mountainous soil in southwestern China and dividing the Se-rich soil threshold.

## Introduction

Selenium (Se) is an essential trace element in the human body, playing a crucial role in maintaining a balance between the antioxidant and oxidative systems^1^. Sufficient Se levels can enhance immune function and prevent many diseases, such as chronic osteoarthropathy (Kashin-Beck disease) and chronic heart disease (Keshan disease)^2,3^. Se is mainly obtained via dietary consumption since it cannot be synthesized by the human body. Therefore, as the foundation of the entire food chain, soil crop systems significantly influence the level of dietary Se intake by humans^4,5^. Crops absorb inorganic Se from the soil and convert it into organic Se for human consumption^6,7^. Therefore, examining Se migration in soil-crop systems and evaluating soil Se supply capacity is necessary to effectively utilize soil Se resources and improve human Se nutrition levels.

A total soil Se content exceeding 0.4 mg·kg^−1^ is considered the threshold of Se-rich soil in China and is used for developing and utilizing Se-rich soil resources in accordance with the national standard DD 2019–10 delimitation and logo for natural Se-enriched land^8,9^. However, studies have shown that even growing in Se-rich areas or Se poisoned areas with high total Se content, there will be low Se contain in crops^10^. Meanwhile, Se as a trace element, is closely related to the geological background in soil content^11^. Furthermore, cultivated land with low total Se content can also produce Se-rich crops due to the influence of several factors on soil Se absorption and plant utilization^12^. On the one hand, the Se accumulation in crops is related to the total Se content in the soil and depends more on the available Se that can be absorbed and utilized by crops^7,13^. On the other hand, physical and chemical soil properties, crop species, and other factors also affect Se absorption by crops^14^. The species of crops significantly influences its ability to absorb and accumulate Se^15^. Thiry et al.^16^ found that the total Se content in vegetables ranges from 51 to 601 mg·kg^−1^, while the total Se content in the grains of staple crops generally remains below 100 mg·kg^−1^. Plants can be categorized into three types based on their capacity to absorb Se, which are accumulator plants (indicator plants), moderate Se-accumulating plants, and low Se-accumulating plants^17^. Additionally, within the same plant, different parts exhibit varying abilities to absorb Se. Generally, non-edible parts of crops tend to absorb more Se than the edible parts^18^. Therefore, when evaluating regional Se resources, researchers should consider the soil Se content and pay attention to the Se content and soil enrichment degree in different crops^18,19^. However, most existing studies on Se-rich soils only evaluate the soil Se threshold standard, ignoring the crop Se content and other environmental factors, which reduces the scientificity and rationality of soil Se resource development and utilization^20^. Determining the environmental consequences of Se in naturally Se-rich locations near agricultural soil systems is currently considered a more scientific method. The relevant research results show variation in the soil Se-rich threshold in different regions. Wang Ying et al.^21^ determined the total Se content threshold of Se-rich soil in northern Ningxia as 0.24 mg·kg^−1^, while Wang Rui et al. revealed the soil Se-rich threshold range in the Qianjiang District of Chongqing by combining soil pH and crop types^20^. Chongqing, Ningxia, Heilongjiang, and other provinces have published categorization standards for local Se-rich soils in response to the discrepancy between Se-rich soils and Se-rich crops. The threshold value of Se-rich land delimitation varies between 0.22 and 0.40 mg·kg^−1^, leading to the incompatibility between Se-rich soils in different regions. Therefore, examining the threshold and factors affecting Se-rich soil in various naturally Se-rich areas is necessary.

Existing researches has revealed a pronounced Se-deficient zone from Northeast to Southwest China, showing a high incidence of related human diseases^4,5,22^. Recent studies have indicated the distribution of large Se-rich soil areas in southwestern China, especially the mountainous regions in southwest Yunnan province, showing both surface soil and crop Se content abundance^23^. However, minimal research is available on the Se-rich resources in this region, and no local standards have been established. This study focuses on Ximeng County in Yunnan province to analyze the spatial distribution characteristics of soil Se, crop Se enrichment coefficient, and influencing factors. Furthermore, the threshold for Se-rich soil is explored according to different crop types to establish a scientific basis for developing Se-rich resources and delineating Se-rich lands in the region.

## Materials and methods

### Study area

Ximeng County is located in southwestern China (99°18′—99°43′ E, 22°25′—22°57′ N) (Figure S1), covering an area of about 1353.57 km^2^. Affected by the warm, humid air in the southwest of the Bay of Bengal, it presents a subtropical marine monsoon climate. The region is located at a height of 1869.9 m with regular precipitation of approximately 2758.3 mm per annum, an average annual temperature of 15.3 °C, and about 2204.7 h of sunlight. The average soil pH is 5.81, which is generally acidic, with an approximate soil organic matter (SOM) content of 37.00 g·kg^−1^. The soil is classified as Ultisols in the USDA Soil Taxonomy^24^. The primary grain crops include rice and maize, while tea, coffee, and nuts represent the main cash crops^25^.

### Soil sample collection

**Figure 1:**
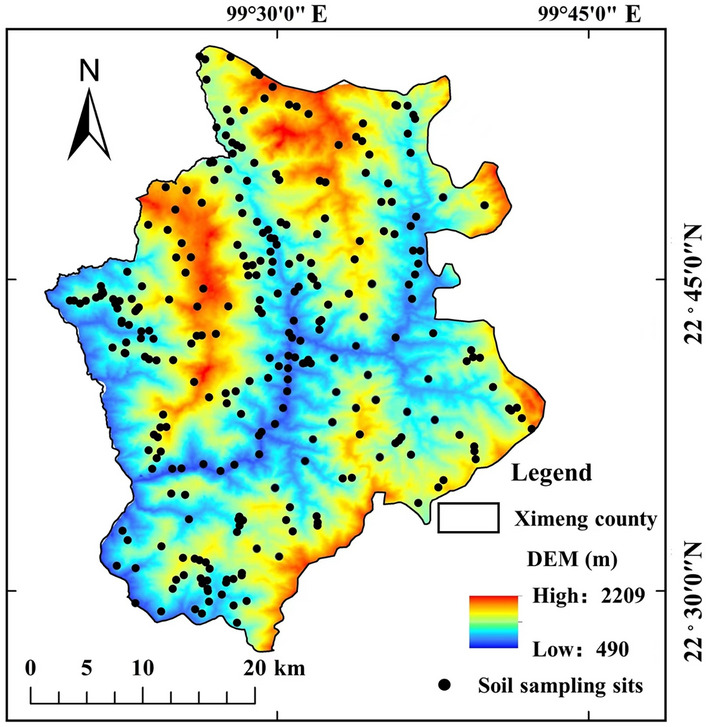
A schematic diagram of the soil sampling points (National catalogue service for geographic information of China, National county-level administrative boundaries shapefile data. https://www.webmap.cn/main.do?method=index. Geospatial data cloud, 30-m resolution digital elevation data. https://www.gscloud.cn/). The map were created using Arc GIS Geographic Information Systems software version 10.2 (Environmental Systems Research Institute Inc, Redlands, Calif. URL: https://www.esri.com/zh-cn/home).

The five-point sampling method was used to collect 275 soil samples at a depth of 0–30 cm in Ximeng County according to the township area, planting structure, landform, and other factors (Fig. 1). The excess soil was discarded via the quartering method, with 1 kg for each mixed sample, while the altitude, longitude, and latitude of the sampling points were recorded.

### Soil sample treatment and analysis

The soil samples were dried naturally in the laboratory, ground, screened (10 mesh) after removing stones and plant residues, and placed in self-sealing bags for testing. The pH of soil in water(1:2.5, w/v) was measured using a pH meter (PHS-3C). The SOM was determined by the potassium dichromate-sulfuric acid titration. The determination of total Se in soil referred to national agricultural industry standards (NY/T 1104–2006)^26^ using the atomic fluorescence spectrometry(AFS 8520, Haiguang Instrument Co., Ltd., Beijing, China). The determination of available Se in soil was conducted in accordance with the national agricultural industry standard (NY/T 3420–2019)^27^ using the hydride generation atomic fluorescence spectrometry(AFS 8520, Haiguang Instrument Co., Ltd., Beijing, China). The pH, SOM, total Se, available Se in the soil samples were determined, as shown in Table S1.

### Crop sample collection

The collection of crop samples was approved by the local government and agricultural departments. The collection process was conducted in accordance with the Agricultural Industry Standards of the People's Republic of China (NY/T 398–2000)^28^ and the local standards (DB51/T 1047–2010)^29^. Based on the agricultural planting structure and soil sampling locations within the study area, 153 samples of the edible parts of mature crops were correspondingly collected (Table 1), including 70 grain samples, such as rice (*Oryza sativa L.*) and maize (*Zea mays L.*), and 83 cash crop samples, such as tea (*Camellia sinensis L.*), nuts (*Macadamia integrifolia*), konjac (*Amorphophallus rivieri*), mushrooms (*Boletus edulis*), buckwheat (*Fagopyrum esculentum*), and coffee (*Coffea arabica L.*). The edible parts of the crops were collected from the same locations as the soil samples, ensuring that each crop sample corresponded directly to its respective soil sampling point. Crop samples ≥ 500 g were placed in self-sealing bags, numbered, and stored for safekeeping.

**Table 1:** Crop sample details.

Crop species	Grain samples		Cash crop samples					
	Rice	Maize	Tea	Nuts	Konjac	Mushrooms	Buckwheat	Coffee
Number of samples	43	27	63	10	4	2	2	2

### Crop sample treatment and analysis

Crop samples were air-dried under natural conditions in a clean, uncontaminated area. The dried samples were then ground to a 60 mesh size using a pulverizer. The Se content in the crops was determined according to the national food safety standard determination of Se in food (GB 5009.93-2017)^30^ using the atomic fluorescence spectrometer (AFS-8520, Haiguang Instrument Co., Ltd., Beijing, China) at a detection limit of 0.006 mg·L^−1^.

### Data analysis

The indexes were described using IBM SPSS Statistics 23, while GS^+^9.0 was employed for semi-variance function fitting, and modeling was achieved via Random Forest model and multiple regression models. The graphs were drawn using ArcGIS 10.2 and Origin 2018.

## Results and discussion

### The characteristics and factors influencing the soil Se content

#### The soil Se content statistics

The statistical results of the Se content in the 275 collected soil samples are shown in Table 2. The total Se content in the soil ranged from 0.056 mg·kg^−1^ to 2.177 mg·kg^−1^, with an average content of 0.535 mg·kg^−1^ and a coefficient of variation of 55.72%. The available Se content in the soil ranged from 0.003 mg·kg^−1^ to 0.325 mg·kg^−1^, with an average content of 0.062 mg·kg^−1^ and a coefficient of variation of 48.90%. The average total Se content in the study area was 1.99-fold that of the background soil Se content value in China (0.269 mg·kg^−1^)^31^ and 1.34-fold that of the global Se content (0.398 mg·kg^−1^)^32^. The available Se content was 6.20 times the average value of the water-soluble soil Se content in China (0.010 mg·kg^−1^)^33^. The coefficient of variation of the total and available Se in the soil was relatively high, indicating uneven spatial content distribution.

**Table 2:** The soil Se content statistics.

	Number of samples	Range/mg·kg^−1^	Mean/mg·kg^−1^	SD/mg·kg^−1^	CV/%
Total Se content	275	0.056–2.177	0.535	0.298	55.72
Available Se content	275	0.003–0.325	0.062	0.030	48.90

The soil was classified according to the total Se content standard^21^ (Table S2), as shown in Figure S2. The deficient, marginal, moderate, high, and excess Se proportions in the soil of the study area were 5.00%, 1.80%, 28.48%, 64.72%, and 0.00%, respectively, indicating Se-rich soil with the potential to develop related resources.

#### Spatial distribution characteristics of the soil Se content

Semi-variance function fitting is used to determine the randomness and structural characteristics of the spatial soil Se distribution pattern^34^. The total and available Se in the Ximeng County soil was subjected to semi-variance function fitting after the outliers were eliminated using the Pauta criterion. The results are shown in Table S3. The Gaussian model was used to determine the total and available Se spatial distribution in the soil, producing determination coefficients of 0.950 and 0.876, respectively, and residual errors of 1.338 × 10^–4^ and 8.567 × 10^–8^, both close to 0. This indicated that the fitting accuracy of the two methods was high, adequately reflecting the spatial structure characteristics of the total and available Se in the Ximeng County soil. The nugget effect values of the total and available Se were 5.00 and 3.87%, respectively. This indicated low spatial variability and a strong spatial correlation between the total and available Se in the soil, mainly regulated by natural factors^15,35^. Furthermore, the total and available Se variation range in the soil was 93.27 km and 82.60 km, respectively, indicating significant spatial autocorrelation.

**Figure 2:**
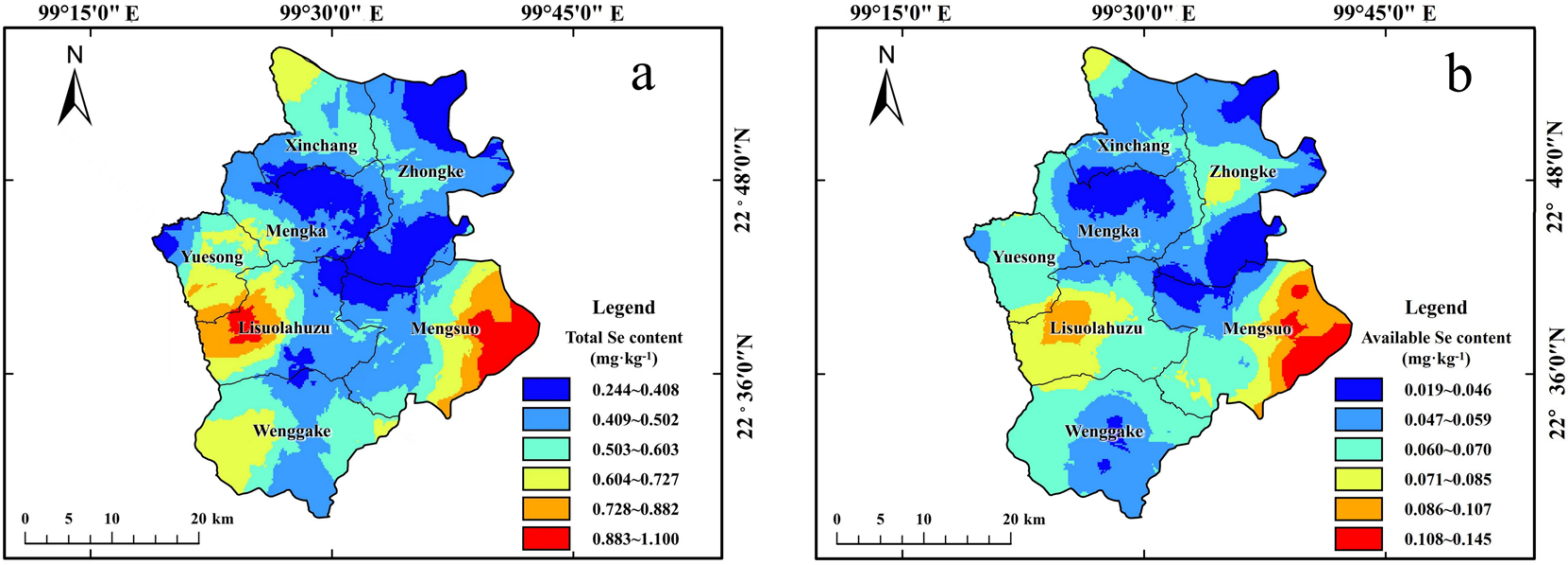
The spatial distribution of the total (**a**) and available (**b**) Se content in the soil (National catalogue service for geographic information of China, National county-level administrative boundaries shapefile data. https://www.webmap.cn/main.do?method=index). The map were created using Arc GIS Geographic Information Systems software version 10.2 (Environmental Systems Research Institute Inc, Redlands, Calif. URL: https://www.esri.com/zh-cn/home).

To further reflect the spatial distribution characteristics of the total and available Se in Ximeng County, ordinary Kriging interpolation was conducted based on semi-variance function fitting. The results are shown in Fig. 2. Ximeng County is high in Se-rich cultivated land resources, distributed in blocks in the middle of Mengka Town, the south of the northeast of Zhongke Town, and at the junction of Lisuolahu Town and Mengsuo Town. The available and total Se distribution in Ximeng County was similar, while the available Se levels were higher in the east of Mengsuo Town and the Midwest of Lisuolahu Town.

The spatial autocorrelation coefficient, Moran's *I,* was used for the global statistical analysis of the soil Se content distribution. The hot spot analysis index (Getis-OrdGi*) was employed for local spatial autocorrelation assessment. The results showed that the soil Se in Ximeng County displayed significant spatial autocorrelation. The total and available soil Se Moran's *I* indexes were 0.79 and 0.66, respectively, *P* < 0.05. The z-scores were 18.20 and 15.50, respectively, which exceeded 1.96, indicating statistical significance^36^.

Figure S3 shows the statistical results of the soil Se content in different strata. The average total Se soil content in the various strata distribution areas appeared in the following order: Devonian (Middle-upper series) 1.038 mg·kg^−1^ > Devonian (Lower series) 0.846 mg·kg^−1^ > Jurassic (Upper Jurassic) 0.740 mg·kg^−1^ > Cretaceous (Lower Cretaceous) 0.710 mg·kg^−1^ > Carboniferous (Southern group) 0.672 mg·kg^−1^ > Cambrian (Wangya formation) 0.527 mg·kg^−1^ > Jurassic (Huakaizuo formation) 0.503 mg·kg^−1^ > Proterozoic (Huiming formation) 475 mg·kg^−1^ > Cambrian (Yungou formation) > Permian granite 0.425 mg·kg^−1^. The average available Se soil content in the different strata distribution areas was in the following order: Devonian (Middle-upper series) 0.111 mg·kg^−1^ > Devonian (Lower series) 0.105 mg·kg^−1^ > Jurassic (Upper Jurassic) 0.095 mg·kg^−1^ > Cretaceous (Lower Cretaceous) 0.081 mg·kg^−1^ > Carboniferous (Southern group) 0.076 mg·kg^−1^ > Jurassic (Huakaizuo formation) 0.062 mg·kg^−1^ > Cambrian (Wangya formation) 060 mg·kg^−1^ > Permian granite 0.057 mg·kg^−1^ > Cambrian (Yungou formation) 0.055 mg·kg^−1^ > Proterozoic (Huiming formation) 0.049 mg·kg^−1^.

These results showed that the Se spatial distribution in the topsoil of the study area was controlled by the stratigraphic distribution characteristics, inferring that the Se content level in the soil of the study area was closely related to the geological background and the type of soil-forming parent material. Due to the high heterogeneity of Se distribution on the Earth's surface, its distribution, migration, and transformation are significantly influenced by environmental geochemical properties and behaviors^37^. Related studies have indicated that the parent material is the primary controlling factor affecting soil Se content^20,37^. Additionally, soil-forming processes, soil physical and chemical properties, and climatic conditions are important factors. The combined influence of these factors determines the Se richness or deficiency in a region's soil, although the relative impact of each factor varies across different regions^19,38^. In the study area, Devonian strata are predominantly exposed as limestone and coal-bearing shale, while Jurassic strata mainly consist of limestone and carbonaceous mudstone. Some researchers suggest that Se-rich soils largely inherit their Se from Se-enriched rocks and coal seams, with shale, clayey sediments, and phosphate rocks generally having higher Se content ^39^. On the one hand, since the coal-bearing black rock series was rich in Se, the soil displayed parent rock inheritance^11^. On the other hand, during the weathering process of the parent rock, the leaching and loss of soluble mineral-rich clay minerals, metal oxides, and organic matter, and adsorption chelation caused Se accumulation on the soil surface^40^. Therefore, the Se content in the soil of the study area is primarily controlled by the parent material, resulting in a stable source of Se. In addition, wet deposition was a primary source of the Se in the soil. The study area was affected by the subtropical marine monsoon climate, with high precipitation levels. Atmospheric deposition may contribute to the Se content in the soil^41^.

#### The factors influencing the available Se content

**Figure 3:**
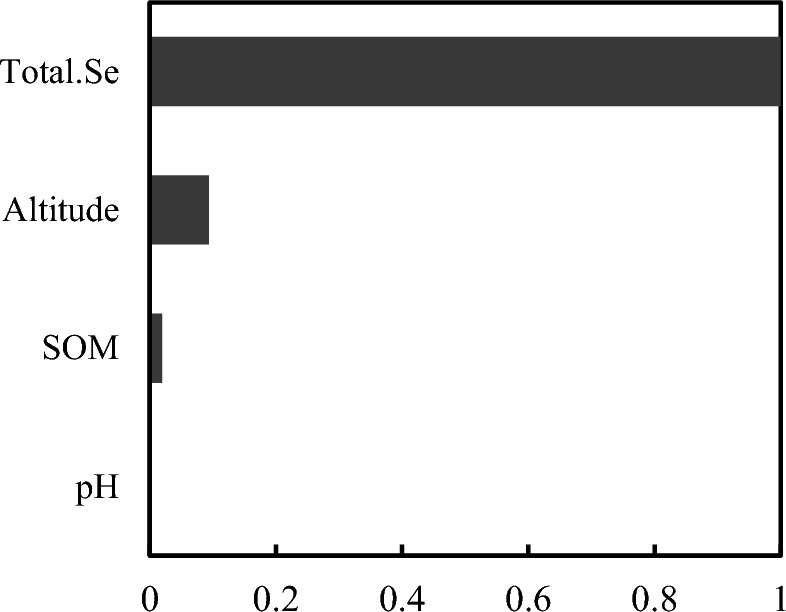
The correlation between the available Se content in the soil and the influencing factors.

The Random Forest model was used for the regression analysis of the total Se content, SOM, pH, altitude, and corresponding available Se levels in the soil. The explanatory degree of the Var% model variable (%Var explained) was 66.42%, indicating a highly significant correlation between the available Se content in the soil and various influencing factors^42,43^. Figure 3 shows the analysis results of the Random Forest model. These results were normalized to show the impact of each influencing factor on the dependent variables from 0 to 1. The total soil Se displayed the closest association with the available Se, followed by altitude and SOM, with pH exhibiting the lowest impact.

The multiple linear regression analysis of the available Se in the soil was conducted using different modeling methods, the independent variables of which were obtained via SPSS software. The results are shown in Table S4. The *R*^*2*^ values after step-by-step, backward, and input modeling were 0.699, 0.704, and 0.705, respectively, with a *Sig* < 0.01, indicating adequate model fitting. Based on the independent variable impact identification via the Random Forest model, the influence of each variable on the available Se content was quantified by constructing a multiple linear regression model. A positive association was evident between the total and available Se content in the soil, while the SOM, pH, and altitude were negatively correlated.

Clarifying the factors influencing the available Se content is essential for targeted bioaugmentation and improving the Se-rich rate of agricultural products. The total Se in soil is the primary source of available Se. playing a regulatory role. Although the total soil Se content has a vital impact, it is not the only factor affecting the available Se content. Various studies have shown that the Se content is closely related to the physicochemical properties of soi^44^. In mountainous areas, the three-dimensional climate is obvious at different altitudes, and the differences in soil parent materials and climatic conditions are the main reasons for the impact of altitude on the available Se content of soil. A study on the Se content in tea garden soil at different altitudes in Fujian Province showed that the Se and available Se levels were significantly higher at medium to high altitudes than at low altitudes^45^, indicating that altitude represented a vital influencing factor. Furthermore, SOM significantly impacts the geochemical behavior of the soil Se, about 80% of which combines with organic matter. In soil rich in organic matter, Se can preferentially enter the low molecular-weight humus components and become fixed in the soil as the inorganic composite state of metal humus^46^. However, this study showed that the SOM was negatively correlated with the available Se content and might be related to the duality of the ecological SOM effect on Se. The ability of SOM to activate soil Se decreased due to the combined impact of other factors, such as soil pH and microbial communities^47^. The correlation between the soil pH and the available Se in the study area was relatively low, showing that this was not the main regulatory factor. This was consistent with the results of Wang et al.^21^ but differed from Liu et al.^48^, who proposed that pH represented the main factor controlling the available Se in soil and that these levels increased at a higher pH. This may be due to single acidic soil in the study area. These results showed that the impact of a single factor on the available Se in the soil cannot be generalized. The influence of the interaction between various factors on soil Se activation requires comprehensive consideration.

### The characteristics and factors influencing the Se content in crops

#### Crop Se content statistics

**Figure 4:**
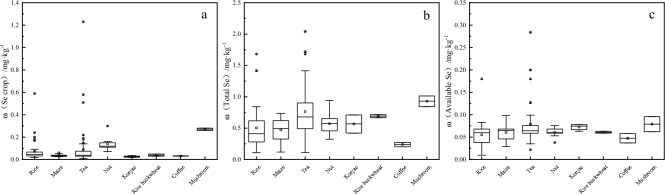
The statistical characteristics of the Se content in the corresponding crop-root soil samples. (**a**) Se content in the crops. (**b**) Total Se content in the crop-root soil. (**c**) Available Se content in the crop-root soil.

The statistical analysis results of the Se content in the crops in the study area are shown in Fig. 4a. The average Se content in rice and maize were 0.073 mg·kg^−1^ and 0.035 mg·kg^−1^, respectively, and 0.090 mg·kg^−1^, 0.141 mg·kg^−1^, 0.026 mg·kg^−1^, 0.040 mg·kg^−1^, 0.032 mg·kg^−1^, and 0.270 mg·kg^−1^ in the tea, nut, konjac, buckwheat, coffee, and mushroom The range of total Se content in the root-soil of each crop is shown in Fig. 4b. The average total Se content in the root soil of rice and maize were 0.518 mg·kg^−1^ and 0.473 mg·kg^−1^, respectively, and 0.765 mg·kg^−1^, 0.570 mg·kg^−1^, 0.567 mg·kg^−1^, 0.692 mg·kg^−1^, 0.240 mg·kg^−1^, and 0.930 mg·kg^−1^ in the tea, nut, konjac, buckwheat, coffee, and mushroom soil. The available Se content range in the root-soil of each crop is shown in Fig. 4c. The average available Se content in the root-soil of rice and maize were 0.055 mg·kg^−1^ and 0.060 mg·kg^−1^, respectively, and 0.079 mg·kg^−1^, 0.061 mg·kg^−1^, 0.073 mg·kg^−1^, 0.061 mg·kg^−1^, 0.048 mg·kg^−1^, and 0.079 mg·kg^−1^ in the tea, nut, konjac, buckwheat, coffee, and mushroom soil.

Based on the corresponding standards delineated by the requirements of Se contents in Se-enriched agro-products (DB5115/T 17-2020)^49^, the Se enrichment rates of the rice and maize samples were 79.54% and 74.07%, respectively, and 15.87%, 100%, 100%, 100%, 100%, and 100% for the tea, nut, konjac, buckwheat, coffee, and wild mushroom samples. Therefore, soil with high total or available Se content does not necessarily render crops Se-rich agricultural products, while soil with low levels may cultivate Se-rich crops^12^. This is not only attributed to the different utilization abilities of Se elements in soil by different crops but also to environmental conditions such as soil properties and climate that can affect Se enrichment^44,50,51^.

#### The crop Se bio-concentration factors

**Figure 5:**
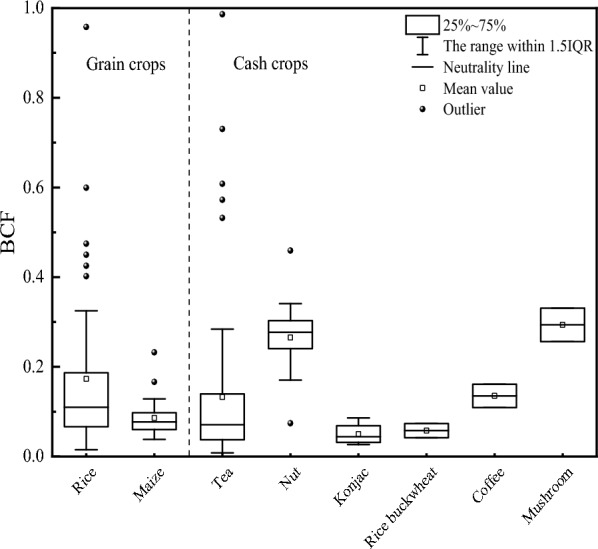
The crop Se BCF.

Bio-concentration factors (BCF) refer to the ratio of the crop sample element content to the corresponding root soil element level, reflecting the absorption and enrichment ability of crops to elements in soil^52^. The BCF of the soil Se in various crops is shown in Fig. 5. The Se BCF of the rice and maize ranged from 0.015 to 0.958 and 0.038 to 0.232, respectively, with averages of 0.173 and 0.086. The Se BCF of the tea, nuts, konjac, buckwheat, coffee, and mushrooms ranged from 0.008 to 0.986, 0.074 to 0.459, 0.027 to 0.086, 0.042 to 0.073, 0.109 to 0.161, and 0.256 to 0.331, respectively, with averages of 0.133, 0.265, 0.050, 0.058, 0.135, and 0.294.

#### The factors influencing the Se BCF of the crops

**Figure 6:**
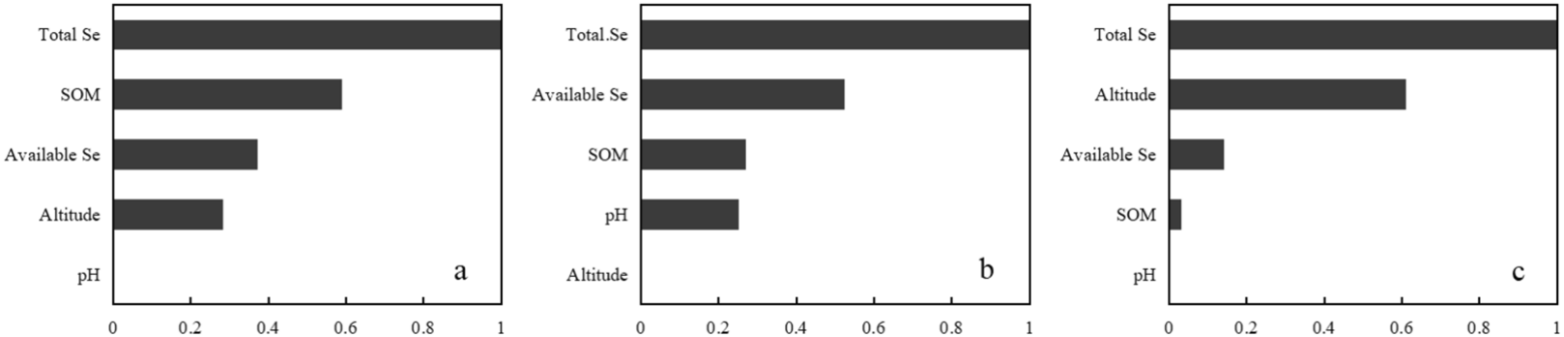
The correlation between the crop Se BCF and the influencing factors. (**a**) Rice with a %Var explained of 20.98%. (**b**) Maize with a %Var explained of 27.53%. (**c**) Tea with a %Var explained of 17.94%.

The study selected crop (rice, maize, and tea) sample sizes larger than 20 to ensure the reliability of the model results. The Random Forest model was used for regression analysis of the total Se content, available Se content, SOM, pH, altitude, and corresponding crop Se content. The Var% (%Var explained) of the variables in the rice, maize, and tea models were 20.98%, 27.53%, and 17.94%, respectively, indicating a significant correlation between the Se content of the three crops and various influencing factors. Figure 6 shows the analysis results of the Random Forest model. These results were normalized to show the impact of each influencing factor on the dependent variables from 0 to 1. As shown in Fig. 6a, the impact order of the various influencing factors on the Se content in the rice was total Se > SOM > available Se > altitude > pH, while the order in the maize samples was total Se > available Se > SOM > pH > altitude (Fig. 6b) and total Se > altitude > available Se > SOM > pH in the tea samples (Fig. 6c).

SPSS was used for multiple regression analysis to obtain the crop absorption model. The results are shown in Table S5. The *R*^*2*^ values of the Se element absorption models for the rice, maize, and tea were 0.403, 0.860, and 0.314, respectively. The *Sig* values were all below 0.01, indicating the reliability of the model fitting results. According to the model equation, the total Se content and altitude in the soil were negatively correlated with the BCF of the three crops. The available Se and pH in the soil were positively correlated with the BCF of the three crops, while the SOM was positively association with the BCF of the maize and the tea and negatively with that of rice.

The Se crop enrichment was influenced by the soil Se content, basic soil properties, and environmental factors, such as altitude^39^. Moreover, the impact and correlation of the same factor on the Se BCF of different crops varied, indicating that crop type represented a vital factor influencing the Se bioavailability^5^. Therefore, Se-enriched soil evaluation should consider the crop species and the specific elemental content in the soil. Relying solely on the soil Se content for assessment can lead to errors.

### Se-rich soil threshold

China is expansive, spanning multiple climatic zones with diverse geological environments and soil types. Consequently, significant differences are evident between the soil Se inception in the various regions. Using a unified total Se soil standard for soil inevitably raises the issue of regional applicability^20^. As shown in Figure S4, using a threshold value of 0.4 mg·kg^−1^ for the total Se content (DD 2019-10)^9^ or a range of 8–20 μg·kg^−1^ for the bioavailable Se content as the definition of Se-rich soil causes significant misjudgment and omission^53^.

The main crops planted in the study area, namely rice, maize, and tea, were selected for examination. The thresholds in the study area were divided according to the Se-rich crop standards provided in the literature (DB5115/T 17-2020)^49^ (0.03 mg·kg^−1^ for rice and maize and 0.1 mg·kg^−1^ for tea). The results are shown in Figure S5, indicating significant misjudgments and omissions in defining Se-rich crops when using a Se-rich soil threshold of 0.4 mg·kg^−1^ (DD 2019-10)^9^ for delineation. The evaluation results of the crops were analyzed after adjusting the Se-rich soil thresholds. The results are shown in Table S6, indicating that the proportion of correct samples increased after the Se-rich soil threshold adjustment. However, the proportion of misjudged rice and maize samples rose as the accuracy increased. Therefore, sample accuracy cannot be the only criterion for evaluation.

**Figure 7:**
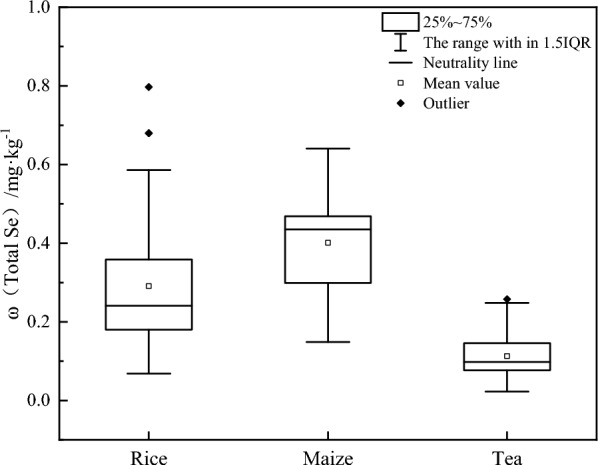
The calculated Se-rich soil threshold results.

The Se-rich threshold of the soil was reverse calculated according to the element absorption model (Table S5), soil Se content detection data, and Se-rich crop standard (DB5115/T 17-2020)^49^. The results are shown in Fig. 7. Based on a rice grain Se enrichment threshold of 0.03 mg·kg^−1^, it is inferred that the total Se content in the soil ranges from 0.07 to 0.79 mg·kg^−1^, with an average content of 0.29 mg·kg^−1^. Based on a maize Se-rich threshold of 0.03 mg·kg^−1^, it is inferred that the total Se content in the soil ranges from 0.15 to 0.64mg·kg^−1^, with an average content of 0.40 mg·kg^−1^. According to a Se-rich threshold of 0.10 mg·kg^−1^ in tea, it is inferred that the total Se content in the soil ranges from 0.02 mg·kg^−1^ to 0.26 mg·kg^−1^, with an average content of 0.11 mg·kg^−1^.

As shown in Table S6 and Fig. 7, using a soil Se-rich threshold of 0.3 mg·kg^−1^ for rice was more appropriate. Although the accuracy was not the highest, the omission and misjudgment rates were relatively low. A soil Se-rich threshold of 0.3 mg·kg^−1^ was suitable for maize, displaying the highest accuracy and fairly low omission and misjudgment rates. Although the accuracy for tea increased at a higher Se enrichment threshold, the model calculation results indicated an average Se content in the tea soil of 0.1 mg·kg^−1^. This could be attributed to the low Se-rich rate of the tea samples in the study area. The edible, tender tea leaves at the top of the tea plant and the shorter growth cycle may be the main reason for the low Se levels. Therefore, the soil Se-rich threshold of the tea remained at 0.4 mg·kg^−1^. Due to the lack of local standards for Se-rich soils in the study area and even in Yunnan Province, this study provides a basis for the threshold classification of the related Se-rich soils, even in southwestern China.

## Conclusion

The study area is a naturally Se-rich region with the potential for developing naturally Se-rich agricultural products. However, the spatial distribution of Se in the soil is uneven and exhibits notable spatial autocorrelation. In particular, the available Se content in the soil is closely associated with total Se and is also influenced by altitude and SOM. Crops' ability to accumulate Se is primarily controlled by the total Se content in the soil, but soil properties and environmental factors also play a significant role in determining Se levels in crops. The model for Se uptake in crops revealed that the Se-rich soil thresholds vary across different crop species. These results indicate that regional Se resource assessments should not only consider the total Se content in the soil but also take into account the soil's physicochemical properties, climatic conditions, and the Se accumulation capacity of different crops.

### Supplementary Information

Supplementary Information.

## Data Availability

Data will be made available on request. Please contact Naiming Zhang (E-mail:zhangnaiming@sina.com), the corresponding author of this article.
