# Supplementary material for: Distribution and soil threshold of selenium in the cropland of southwest mountainous areas in China
Source: Sci Rep. 2024 Jul 23;14:16923. doi: 10.1038/s41598-024-67450-7 (PMC11266564; doi:10.1038/s41598-024-67450-7)
Supplement: Supplementary file 1 — Supplementary Information. [file 41598_2024_67450_MOESM1_ESM.docx]

**Distribution and Soil Threshold of Selenium in the Cropland of Southwest Mountainous Areas in China**

**Supplementary materials**

# **MATERIAL AND METHODS**

## Study area


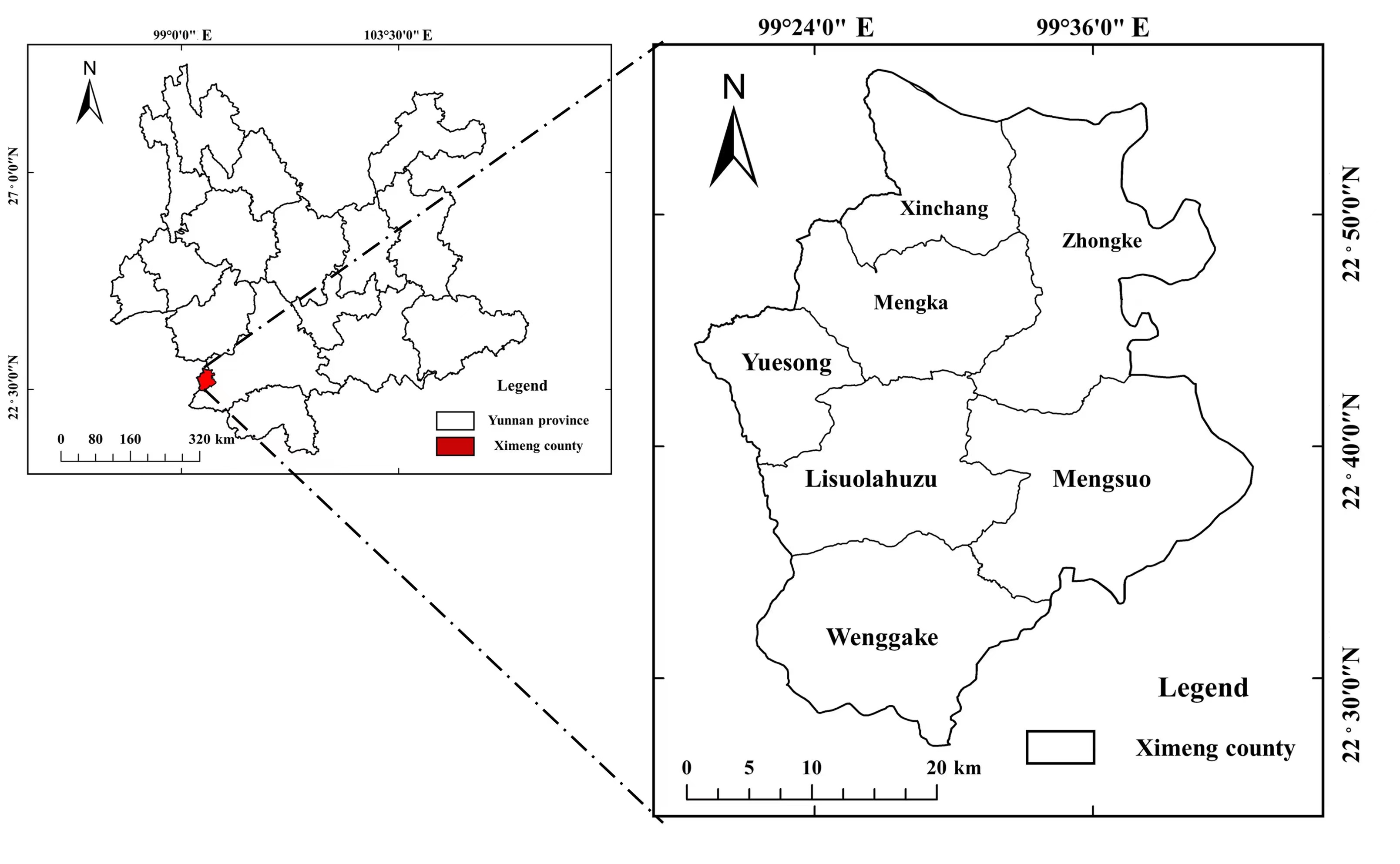


Figure S1. An overview of the research area (National catalogue service for geographic information of China, National County-level Administrative Boundaries Shapefile Data. https://www.webmap.cn/main.do?method=index). The map were created using Arc GIS Geographic Information Systems software version 10.2 (Environmental Systems Research Institute Inc, Redlands, Calif. URL: https://www.esri.com/zh-cn/home).

## Soil sample treatment and analysis

The detection limits of the analytical methods were consistent with the Specification of Land Quality Geochemical Assessment (DZ/T 0295-2016) requirements.

Table S1. Soil sample index analytical methods

| **Index** | **Method** | **Detection limit** |
| --- | --- | --- |
| pH | ISE | 0.1 |
| SOM, g·kg^-1^ | VOL | 0.05 |
| Total Se, mg·kg^-1^ | AFS | 0.01 |
| Available Se, mg·kg^-1^ | AFS | 0.006 |

# RESULTS AND DISCUSSION

## The characteristics and factors influencing the soil Se content

### The soil Se content statistics

Table S2. The soil total Se content classification standards

| Grade | Deficient | Marginal | Moderate | High | Excess |
| --- | --- | --- | --- | --- | --- |
| Se / mg·kg^-1^ | ≤0.125 | 0.125~0.175 | 0.175~0.4 | 0.4~3.0 | ＞3.0 |


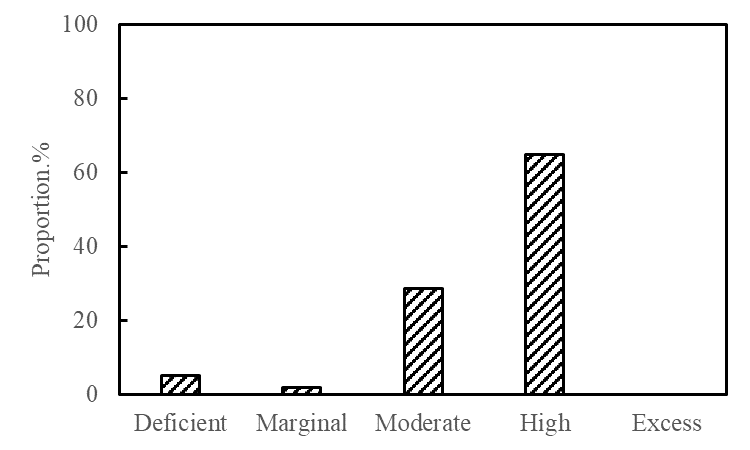


Figure S2. The soil total Se content grade statistics

### Spatial distribution characteristics of the soil Se content

Table S3. The semi-variance function model and corresponding parameters of the total and available Se content in the soil

|  | Model | Nugget | Partial sill | Nugget effect/% | Rang/km | Fitting coefficient | Residual |
| --- | --- | --- | --- | --- | --- | --- | --- |
| Total Se | Spherical model | 0.002 | 0.067 | 2.99 | 61.10 | 0.877 | 3.303×10^-4^ |
|  | Exponential model | 0.009 | 0.115 | 7.83 | 183.30 | 0.863 | 3.678×10^-4^ |
|  | Gaussian model | 0.009 | 0.180 | 5.00 | 93.27 | 0.950 | 1.338×10^-4^ |
|  | Linear model | 0.002 | 0.045 | 4.44 | 28.03 | 0.895 | 2.839×10^-4^ |
| Available Se | Spherical model | 1.00×10^-6^ | 9.26×10^-4^ | 0.11 | 61.10 | 0.735 | 1.829×10^-7^ |
|  | Exponential model | 1.00×10^-6^ | 1.57×10^-3^ | 0.06 | 183.30 | 0.715 | 1.984×10^-7^ |
|  | Gaussian model | 8.40×10^-5^ | 2.17×10^-3^ | 3.87 | 82.60 | 0.876 | 8.567×10^-8^ |
|  | Linear model | 1.00×10^-6^ | 9.90×10^-4^ | 0.10 | 9.99 | 0.760 | 2.159×10^-6^ |

### Spatial distribution characteristics of the soil Se content


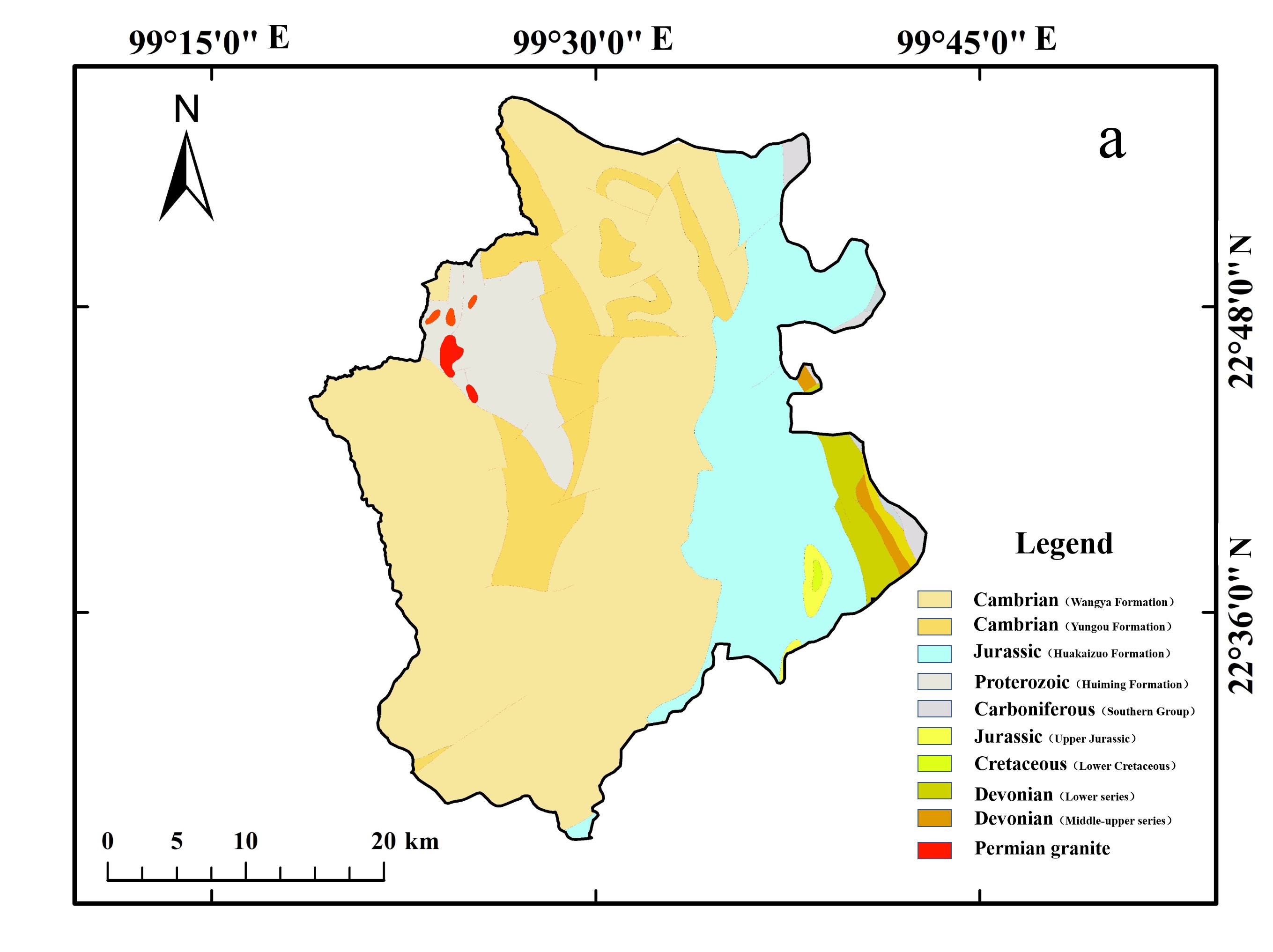

Figure S3. Spatial distribution of strata in Ximeng County. (a) The schematic diagram of the stratigraphic distribution in the study area. (b) The statistical result of selenium content in soils from different strata. (China Geological Survey, National Geological Library. https://www.ngac.cn/125cms/c/qggnew/index.htm). The map were created using Arc GIS Geographic Information Systems software version 10.2 (Environmental Systems Research Institute Inc, Redlands, Calif. URL: https://www.esri.com/zh-cn/home).

### Factors influencing the available Se content

Table S4. The multiple linear regression equations of the factors influencing the available Se using different modeling approaches

| Modeling method | Equation | *R^2^* | *Sig.* |
| --- | --- | --- | --- |
| Step by step | Available Se = 0.096 Total Se + 0.010 | 0.699 | 0.000 |
| Backward | Available Se = 0.098 Total Se － (3.589E－6) Altitude + 0.013 | 0.704 | 0.000 |
| Input | Available Se = 0.098 Total Se － (3.703E－6) Altitude － (2.133E－5) SOM － 0.001 pH + 0.022 | 0.705 | 0.000 |

## The characteristics and factors influencing the Se content in crops

### The factors influencing the Se BCF of the crops

Table S5. The multiple linear regression equations of the factors influencing the crop Se BCF using different modeling approaches

| Crop types | Element absorption model | *R^2^* | *Sig.* |
| --- | --- | --- | --- |
| Rice | lg BCF = － 0.726 lg Total Se － 0.210 lg SOM + 0.177 lg Available Se － 0.270 lg Altitude + 0.809 lg pH － 0.421 | 0.403 | 0.001 |
| Maize | lg BCF = － 0.769 lg Total Se + 0.249 lg Available Se + 0.065 lg SOM + 0.507 lg pH － 0.104 lg Altitude － 1.237 | 0.860 | 0.000 |
| Tea | lg BCF = － 0.690 lg Total Se － 1.748 lg Altitude + 0.366 lg Available Se + 0.336 lg SOM + 0.154 lg pH + 5.184 | 0.314 | 0.001 |

## Se-rich soil threshold

Figure S4. The concordance analysis of the total and available Se content in the soil samples

Figure S5. Se-rich soil threshold. (a) The synergistic relationship between the Se levels in the rice and maize and the total Se content in the soil (n=71). (b) The synergistic relationship between the Se level in the tea and the total Se content in the soil (n=63).

Table S6. The statistical results of the crops at different Se-rich soil thresholds

| Crop types | Se-rich Soil Thresholds/mg·kg^-1^ | The proportion of correct samples/% | The proportion of missing samples/% | The proportion of misjudged samples/% |
| --- | --- | --- | --- | --- |
| Rice | 0.1 | 35 (79.55%) | 0 (0%) | 9 (20.45%) |
|  | 0.2 | 33 (75.00%) | 3 (6.82%) | 8 (18.18%) |
|  | 0.3 | 32 (72.73%) | 7 (15.91%) | 5 (11.36%) |
|  | 0.4 | 27 (61.36%) | 14 (31.82%) | 3 (6.82%) |
|  | 0.5 | 24 (54.54%) | 17 (38.64%) | 3 (6.82%) |
| Maize | 0.1 | 20 (74.07%) | 0 (0%) | 7 (25.93%) |
|  | 0.2 | 22 (81.48%) | 0 (0%) | 5 (18.52%) |
|  | 0.3 | 22 (81.48%) | 1 (3.70%) | 4 (14.81%) |
|  | 0.4 | 18 (66.67%) | 6 (22.22%) | 3 (11.11%) |
|  | 0.5 | 16 (59.26%) | 9 (33.33%) | 2 (7.41%) |
| Tea | 0.1 | 10 (15.87%) | 0 (0%) | 53 (84.13%) |
|  | 0.2 | 15 (23.81%) | 0 (0%) | 48 (76.19%) |
|  | 0.3 | 18 (28.57%) | 0 (0%) | 45 (71.43%) |
|  | 0.4 | 22 (34.92%) | 0 (0%) | 41 (65.08%) |
|  | 0.5 | 26 (41.27%) | 0 (0%) | 37 (58.73%) |
|  | 0.6 | 34 (53.97%) | 0 (0%) | 29 (46.03%) |
